# REHUNT: a reliable and open source package for restriction enzyme hunting

**DOI:** 10.1186/s12859-018-2168-4

**Published:** 2018-08-10

**Authors:** Yu-Huei Cheng, Jiun-Jian Liaw, Che-Nan Kuo

**Affiliations:** 1 Department of Information and Communication Engineering, Chaoyang University of Technology, Taichung, Taiwan; 2 Department of Business Administration, CTBC Business School, Tainan, Taiwan

## Abstract

**Background:**

Restriction enzymes are used frequently in biotechnology. However, manual mining of restriction enzymes is challenging. Furthermore, integrating available restriction enzymes into different bioinformatics systems is necessary for many biotechnological applications, such as polymerase chain reaction-restriction fragment length polymorphism (PCR-RFLP). Thus, in the present study, we developed the package REHUNT (Restriction Enzymes HUNTing), which mines restriction enzymes from the public database REBASE using a series of search operations.

**Results:**

REHUNT is a reliable and open source package implemented in JAVA. It provides useful methods and manipulations for biological sequence analysis centered around restriction enzymes contained in REBASE. All available restriction enzymes for the imported biological sequences can be identified by REHUNT. Different genotypes can be identified using PCR-RFLP based on REHUNT for single nucleotide polymorphism (SNP), mutations, and the other variations. REHUNT robustly recognizes multiple inputs with different formats, e.g. regular DNA sequences, variation-in-sequence indicated by IUPAC code, as well as variation-in-sequence indicated by dNTPs format. Variations including di-, tri-, and tetra-allelic types and indel formats are also acceptable. Furthermore, REHUNT provides classified restriction enzymes output, including IUPAC and general sequence types, as well as commercial and non-commercial availabilities. REHUNT also enables analysis for high throughput screening (HTS) technologies.

**Conclusions:**

REHUNT is open source software with GPL v3 license and can be run on all platforms. Its features include: 1) Quick restriction enzymes search throughout a sequence based on the Boyer-Moore algorithm; 2) all available restriction enzymes provided and regularly updated from REBASE; 3) an open source API available of integrating all types of bioinformatics systems and applications; 4) SNP genotyping available for plant and animal marker-assisted breeding, and for human genetics; and 5) high throughput analysis available for Next Generation Sequencing (NGS). REHUNT not only to effectively looks for restriction enzymes in a sequence, but also available for SNP genotyping. Furthermore, it can be integrated into other biological and medical applications. REHUNT offers a convenient and flexible package for powerful restriction enzymes analyses in association studies, and supports high throughput analysis. The source codes and complete API documents are available at SourceForge: https://sourceforge.net/projects/rehunt/, GitHub: https://github.com/yuhuei/rehunt, and at: https://sites.google.com/site/yhcheng1981/rehunt.

## Background

Restriction fragment length polymorphism (RFLP) is a useful molecular technique that exploits genetic variants and mutations in homologous DNA. It is based on endonuclease cleavage and is relatively inexpensive for genotyping [1]. Many laboratories use polymerase chain reaction (PCR)-RFLP to rapidly detect point mutations after the genomic sequences are amplified, especially in single nucleotide polymorphism (SNP) genotyping [2–7]. PCR-RFLP has become a convenient method for mutation detection because of its simplicity, low cost, and accuracy, as well as it is utility in small basic research studies of complex genetic diseases. So far, more than 47,900 studies that have used RFLP (not the acronym, but the full term) have been published and indexed in NCBI PubMed (https://www.ncbi.nlm.nih.gov/pubmed/?term="restriction+fragment+length+polymorphism"). These records continue to grow. Specific restriction enzymes in RFLP can discriminate variants and mutations; therefore, finding the correct restriction enzymes is extremely important.

REBASE (http://rebase.neb.com/rebase/) is a well-known database containing information about restriction enzymes, including their recognition and cleavage sites, isoschizomers, neoschizomers, commercial availability, methylation sensitivity, and crystal and sequence data [8]. REHUNT provides a comprehensive and complete resource for researchers. Furthermore, two web servers are particularly valuable for searching restriction enzyme sites. One is Webcutter, the other is NEBcutter [9]. Both identify restriction enzymes that cleave the input DNA sequence. To study or discover genotype variations, SNP Cutter [1] and SNP-RFLPing [10, 11] are useful to identify restriction enzymes that cleave specified SNP sites. The above web servers all combine with REBASE to provide potential restriction enzymes. Although these web-servers are useful to molecular researchers for identifying restriction enzymes, they are aimed at understanding specific functions (or functional moieties), and are thus limited for advanced researchers. Furthermore, they lack periodical maintenance, which limits their functionality.

In this study, we describe a package, termed REHUNT (Restriction Enzymes HUNTing), which is used to extract valuable information to analyze DNA sequences that have variations within their restriction enzyme recognition sequences. REHUNT is free and open source with a GPL v3 release license and is implemented in JAVA. It can be efficiently used to develop all kinds of built-in methods, such as PCR-RFLP. REHUNT has been successfully applied to natural PCR-RFLP primer design for SNP genotyping [12].

## Implementation

REHUNT is mainly implemented by text retrieval technology. The well-known restriction enzyme database REBASE (http://rebase.neb.com/rebase/) [8] is downloaded and integrated into REHUNT. A time-saving exact string matching algorithm, i.e. Boyer-Moore [13], is applied to REHUNT to enhance its ability to search for restriction enzymes. To explain why we chose the Boyer-Moore string matching algorithm instead of finite automatons or deterministic methods, we have provided comparisons of the time complexities among the existing string matching methods including bitap [14], BNDM (Backward Non-Deterministic Dawg Matching) [15], BOM (Backward Oracle Matching) [16], Boyer-Moore, KMP (Knuth-Morris-Pratt) [17], Naïve [18], and Rabin-Karp [19] as shown in Table 1. From Table 1, we can see that although the Boyer-Moore string matching algorithm has slightly higher constant *k* of preprocessing time compared with other existing string matching methods, except Bitap, it has the fastest string matching time. Furthermore, the Boyer-Moore string matching algorithm has been the standard benchmark in the practical string search literature [20].

**Table 1:** Comparisons of time complexity among existing string matching methods

String matching method	Preprocessing time	String matching time
Bitap	Θ(*m* + *k*)	O(*mn*)
BNDM	O(*m*)	O(*n*)
BOM	O(*m*)	O(*n*)
Boyer-Moore	Θ(*m* + *k*)	Best: Ω(*n*/*m*) Worst: O(*mn*)
KMP	Θ(*m*)	Θ(*n*)
Naïve	0	Θ(*nm*)
Rabin-Karp	Θ(*m*)	Average: Θ(*n* + *m*) Worst: Θ((*n* − *m*)*m*)

*m* is the length of the enzyme recognition sequence; *n* is the length of the searchable sequence, and *k* is the size of the symbols of the enzyme recognition sequence; notations O, Ω, and Θ are time complexities that represent the asymptotic times of the algorithms

### Database description

REHUNT uses REBASE in its original text format to facilitate version updating. The restriction enzymes data in REHUNT are available from “All Enzymes (parsed references) (parsrefs)” at http://rebase.neb.com/rebase/rebase.files.html. Currently, REHUNT is released as version 1.2 which uses REBASE version709 with the latest update as of Aug 28 2017.

### Design and analysis

**Fig. 1:**
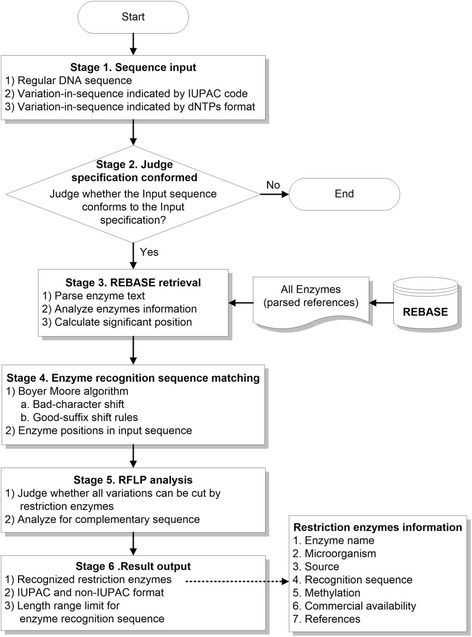
Analysis flowchart of REHUNT

REHUNT uses Object-Oriented Programming (OOP) to develop the open source package. The analysis flowchart of REHUNT is clearly described in Fig. 1. There are six stages including: stage 1. Sequence input; stage 2. Judge specification conformed; stage 3. REBASE retrieval; stage 4. Enzyme recognition sequence matching; stage 5. RFLP analysis; and stage 6. Result output, which is used to perform the operations of REHUNT. In stage 1, users prepare their sequence and input them into REHUNT according to the input specification, including 1) regular DNA sequence, 2) variation-in-sequence indicated by IUPAC code, and 3) variation-in-sequence indicated by dNTPs format. In stage 2, REHUNT judges whether the input sequence conforms to the input specification. If the input sequence does not conform to the input specification, the program will be finished, otherwise REHUNT continues to the next stage. In stage 3, all restriction enzymes are retrieved from REBASE via three operations: 1) Parse enzyme text; 2) analyze enzyme information; and 3) calculate significant position. After all restriction enzymes are loaded into the memory, stage 4 will be performed. Stage 4 executes enzyme recognition sequence matching using the Boyer-Moore algorithm which employs bad-character shift and good-suffix shift rules for the input sequence and all enzyme recognition sequences. Enzymes positions in the input sequence are thus obtained effectively. In stage 5, RFLP analysis can be performed by judging whether all variations can be cut by the restriction enzymes. Furthermore, it also analyzes the complementary sequence. Finally, in stage 6, the results are displayed, containing the recognized restriction enzymes (including enzyme name, microorganism, source, recognition sequence, methylation, commercial availability, and references). Furthermore, the output also provides IUPAC and non-IUPAC format and length range limits for the enzyme recognition sequences. Thus, REHUNT consists of a series of methods related to calculations, searches, manipulations and classification of restriction enzymes in sequences with variations. The calculations performed by REHUNT are 1) restriction enzymes information retrieval from REBASE; 2) exact string matching for the enzyme recognition sequence, and 3) judgment of RFLP for specific variations. The searches performed by REHUNT are 4) restriction enzyme information search, and 5) available restriction enzymes search for RFLP. The manipulations of REHUNT are 6) detection of the existing variations; 7) transformation of sequence variation between [dNTP1/dNTP2/…/dNTP4] and the IUPAC format; 8) combination of sequences with different variations; and 9) retrieval of variation flanking sequences. Finally, the classifications of REHUNT are 10) commercial and non-commercial enzymes, and 11) composite IUPAC and general enzymes. The above methods are described in detail in the following text.

#### Restriction enzymes information retrieval from REBASE

To obtain the available restriction enzymes, we use text retrieval technology to mine the information from the original plain text of REBASE. The text retrieval technology is based on text parsing, specific text location, and significant position calculations. The retrieval information includes enzyme name, microorganism, source, recognition sequence, methylation, commercial availability, and references. The desired retrieval information is stored in an array gradually throughout the entire text of REBASE.

#### Exact string matching for the enzyme recognition sequence

**Fig. 2:**
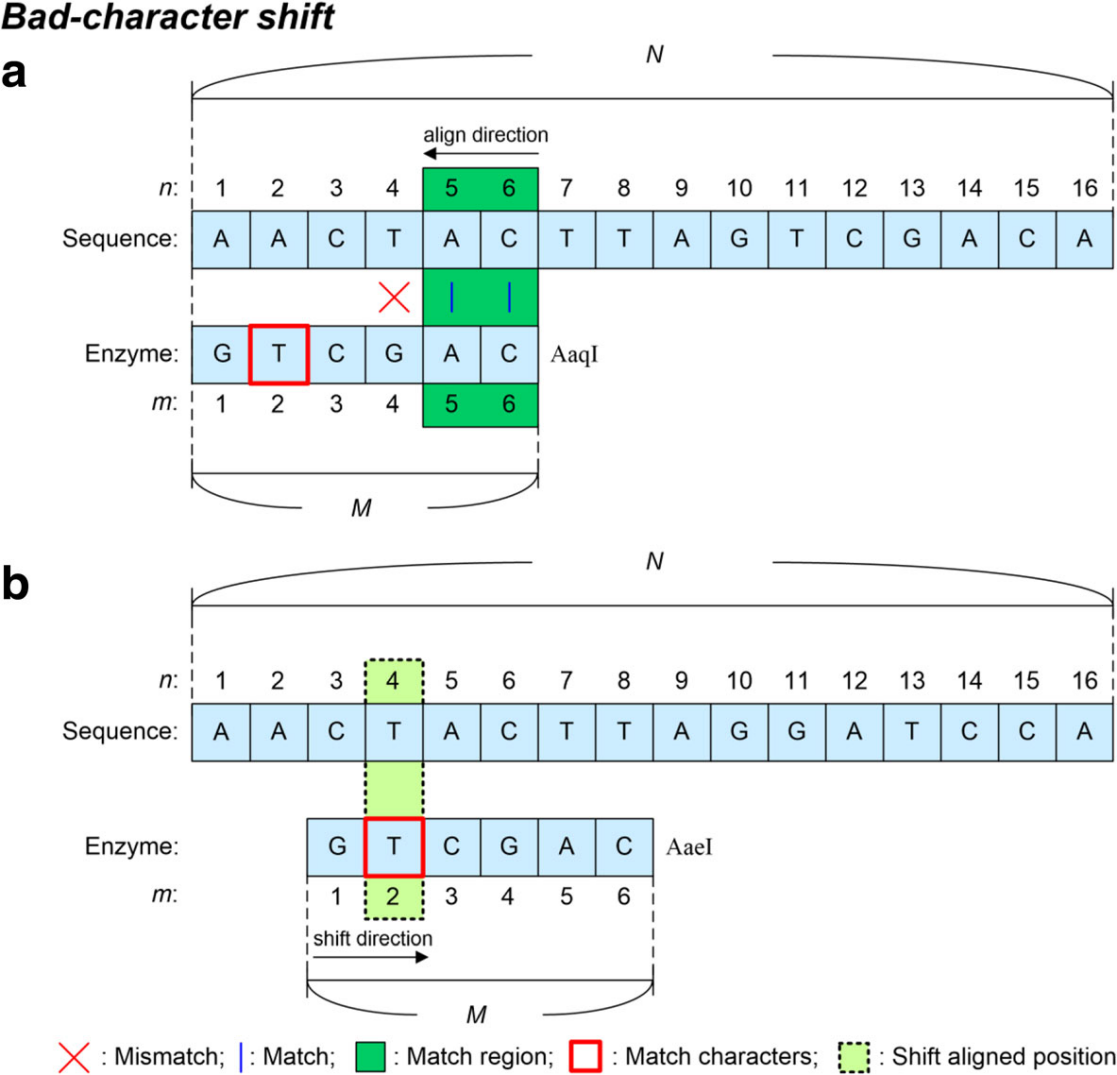
Bad-character shift rule. *N* is the length of the input sequence and *M* is the length of the enzyme recognition sequence. Sequence (*n*) and Enzyme (*m*) represent the nucleotide at the *n*-th position in the input sequence and the nucleotide at the *m*-th position in the enzyme recognition sequence, respectively. **a** An enzyme recognition sequence is aligned from right to left: Sequence (6) = Enzyme (6), Sequence (5) = Enzyme (5), but Sequence (4) ≠ Enzyme (4). **b** The Enzyme window is moved and Enzyme (2) is aligned to Sequence (4)

REHUNT implements an efficient Boyer-Moore algorithm [13] to enhance its search capability to identify recognition sites. In the Boyer-Moore algorithm, both the bad-character shift and the good-suffix shift rules are used. Figure 2 describes the processes of the shift rules. Here, we suppose that *N* is the length of the input sequence and *M* is the length of the enzyme recognition sequence. Sequence (*n*) and Enzyme (*m*) represent the nucleotide at the *n*-th position in the input sequence and the nucleotide at the *m*-th position in the enzyme recognition sequence, respectively. Sequence (*n*_*s*_, *n*_*e*_) and Enzyme (*m*_*s*_, *m*_*e*_) represent nucleotides from the *n*_*s*_-th position to the *n*_*e*_-th position in the input sequence, and nucleotides from the *m*_*s*_-th position to the *m*_*e*_-th position in the enzyme recognition sequence, respectively. As shown in Fig. 2a, the enzyme recognition sequence is aligned to the sequence start from its rightmost to its leftmost position. The aligned condition is Enzyme (6) = Sequence (6), Enzyme (5) = Sequence (5), but Enzyme (4) ≠ Sequence (4). When the aligned character is mismatched, the algorithm searches from the left position of the mismatch position, i.e. Enzyme (4), to the rightmost position of the enzyme recognition sequence to find the same mismatch character using the bad-character shift rule, i.e. Enzyme (2) for Enzyme (2) = Sequence (4). At this stage, the bad-character shift rule then moves the enzyme window and aligns Enzyme (2) to Sequence (4) as shown in Fig. 2b. The alignment of the enzyme recognition sequence then starts again from right to left.

**Fig. 3:**
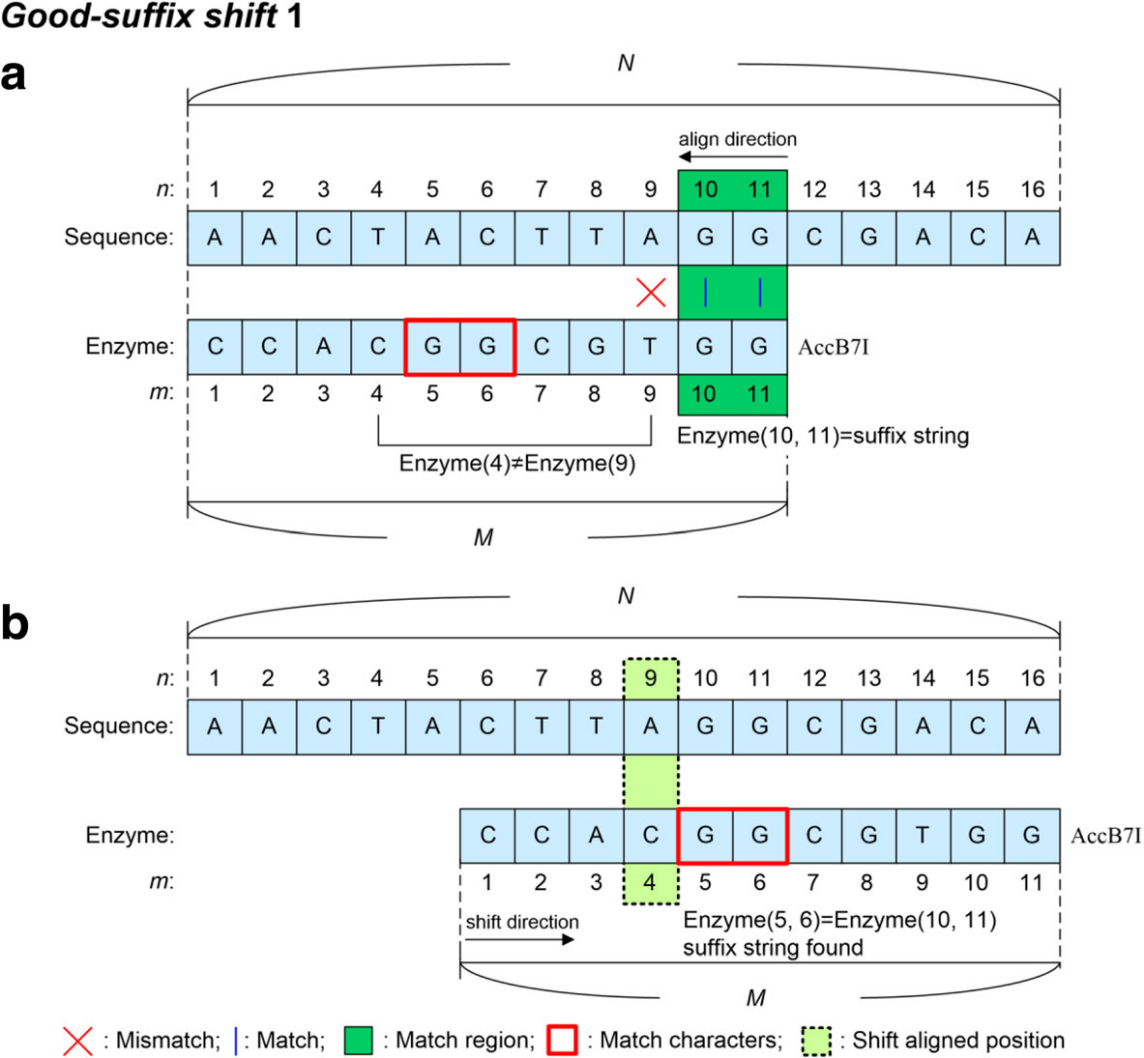
Good-suffix shift 1 rule. *N* is the length of the input sequence and *M* is the length of the enzyme recognition sequence. Sequence (*n*) and Enzyme (*m*) represent the nucleotide at the *n*-th position in the input sequence and the nucleotide at the *m*-th position in the enzyme recognition sequence, respectively. Enzyme (*m*_*s*_, *m*_*e*_) represents nucleotides from the *m*_*s*_-th position to the *m*_*e*_-th position in the enzyme recognition sequence. **a** Enzyme recognition sequence is aligned from right to left: Enzyme (11) = Sequence (11), Enzyme (10) = Sequence (10), but Enzyme (9) ≠ Sequence (9). **b** Enzyme (5,6) is the suffix string of the Enzyme found, and Enzyme (4) ≠ Enzyme (9). The Enzyme window is then moved and Enzyme (4) is aligned to Sequence (9)

**Fig. 4:**
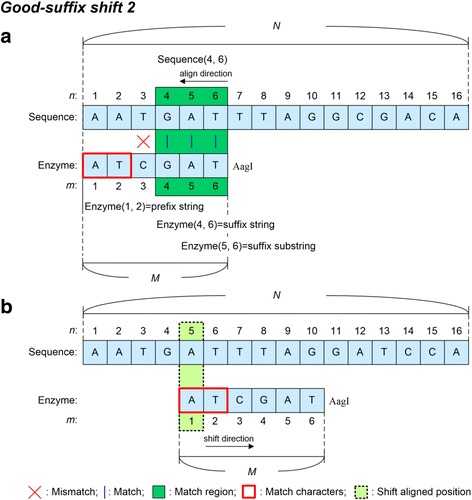
Good-suffix shift 2 rule. *N* is the length of the input sequence and *M* is the length of the enzyme recognition sequence. Sequence (*n*) and Enzyme (*m*) represent the nucleotide at the *n*-th position in the input sequence and the nucleotide at the *m*-th position in the enzyme recognition sequence, respectively. Sequence (*n*_*s*_, *n*_*e*_) and Enzyme (*m*_*s*_, *m*_*e*_) represent nucleotides from the *n*_*s*_-th position to the *n*_*e*_-th position in the input sequence and nucleotides from the *m*_*s*_-th position to the *m*_*e*_-th position in the enzyme recognition sequence, respectively. **a** Enzyme (3) mismatches Sequence (3), Enzyme (4, 6) is the suffix string of the enzyme recognition sequence, and prefix string Enzyme (1, 2) matches the suffix substring Enzyme (5, 6). **b** The Enzyme window is moved and Enzyme (1) is aligned to Sequence (5)

The good-suffix shift rule is divided into two processes, i.e. the good-suffix shift 1 and the good-suffix shift 2. The process for good-suffix shift 1 is described in Fig. 3. In Fig. 3a, the enzyme recognition sequence is aligned starting from its rightmost to its leftmost position. The aligned condition is Enzyme (11) = Sequence (11), Enzyme (10) = Sequence (10), but Enzyme (9) ≠ Sequence (9). When the aligned character is mismatched, the algorithm searches from the left position of the mismatch position, i.e. Enzyme (9), to the rightmost of the enzyme recognition sequence using Good-suffix shift 1 to find the suffix string of the Enzyme, i.e. Enzyme (10, 11), and the right character of the found Enzyme suffix string cannot be the same as the mismatch character. As shown in Fig. 3a, Enzyme (5,6) is the suffix string found and Enzyme(4) ≠ Enzyme (9). The good-suffix shift 1 rule then moves the Enzyme window and aligns Enzyme (4) to Sequence (9), as shown in Fig. 3b. However, if the suffix string cannot be found in Enzyme and the prefix string is the suffix substring of the suffix string of the enzyme recognition sequence, good-suffix shift 2 is performed. Figure 4a shows that Enzyme (3) mismatches Sequence (3), Enzyme (4, 6) is the suffix string of the enzyme recognition sequence, and prefix string Enzyme (1, 2) matches the suffix substring of Enzyme (5, 6), i.e. Enzyme (1, 2) = Enzyme (5, 6) = Sequence (5, 6). Therefore, the good-suffix shift 2 moves the Enzyme window and aligns Enzyme (1) to Sequence (5), as shown in Fig. 4b. The alignment of the enzyme recognition sequence then continues from right to left.

#### Judgment of RFLP for specific variations

RFLP analysis for SNPs, mutations, and other variations is available in REHUNT. To acquire available restriction enzymes that specifically recognize these variations, sequences with different alleles in a variation are first respectively processed to obtain their encoded restriction enzyme recognition sites. After that, these restriction enzymes sites in different sequences with different variations are compared with each other, and duplicated restriction enzymes are eliminated. The reserved restriction enzymes sites are then compared with each other to obtain the specific restriction enzymes.

#### Restriction enzyme information search

REHUNT provides two types of restriction enzyme information searches. One is the search based on the enzyme name, and the other is the search based on the enzyme recognition sequence. Developers can use the enzyme name and the recognition sequence to find the corresponding restriction enzyme information array. The restriction enzyme information array contains the enzyme name, microorganism, source, recognition sequence, methylation, commercial availability, and references. To inherit the advantages of OOP, every elements of the restriction enzyme in the array corresponds to an intuitive accessed method.

#### Available restriction enzymes search for RFLP

**Fig. 5:**
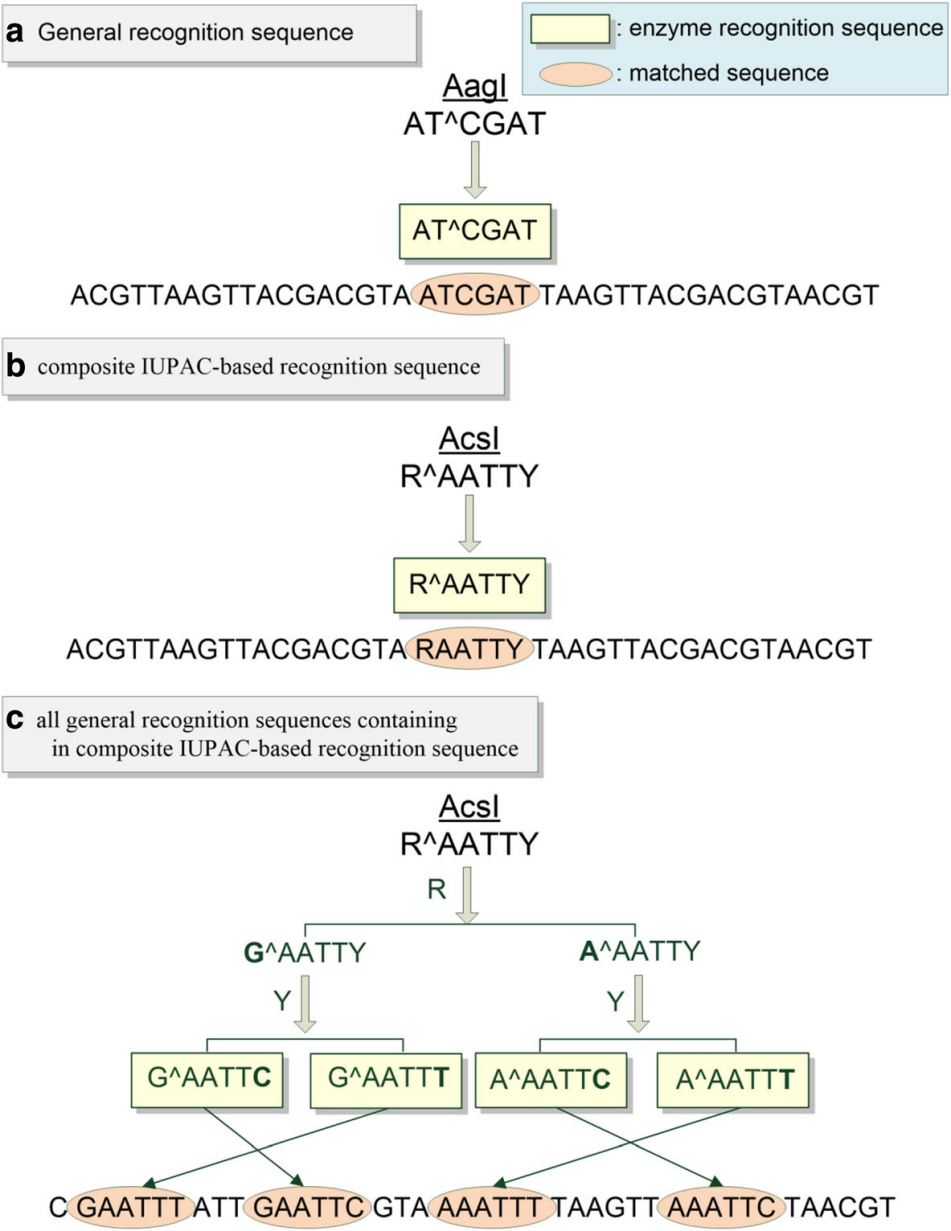
REHUNT searches all recognition sequences containing the composite IUPAC-based recognition sequence for the restriction enzyme

In RFLP, restriction enzymes are provided to recognize the type of variations. REHUNT searches all available restriction enzymes from REBASE by the method described in 2) Exact string matching for the enzyme recognition sequence. Both the general recognition sequence (i.e., only ‘A’, ‘T’, ‘C’, and ‘G’ nucleotide) (Fig. 5a) and the composite IUPAC-based recognition sequence (i.e., ‘R’, ‘Y’, ‘S’, ‘W’, ‘K’, ‘M’, ‘B’, ‘D’, ‘H’, ‘V’, and ‘N’) (Fig. 5b) for restriction enzymes are available for search in REHUNT. Furthermore, all general recognition sequences containing in a composite IUPAC-based recognition sequence (Fig. 5c) are also available for an advanced searched in REHUNT.

#### Detection of the existing variations

REHUNT provides convenient methods to detect if a sequence contains variations and determines their positions. When a sequence is detected to contain variations, many investigations can be performed, such as Human disease/cancer studies, drug development, SNP detection, genetic behavior, and environmental influences.

#### Transformation of sequence variation between [dNTP1/dNTP2] and IUPAC format

All variations [dNTP1/dNTP2/…/dNTP4] within a sequence can be transformed into the composite IUPAC format and vice versa. The transformation helps the nucleotides to correspond to approximate positions. Thus, REHUNT represents a basic and useful method for flexible applications.

#### Combination of sequences with different variations

**Fig. 6:**
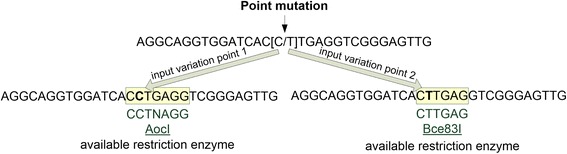
REHUNT provides a combination of sequences in different input variations to search for restriction enzymes

REHUNT is capable to separating all input variations in a point mutation to generate multiple sequences. The multiple sequences can be used to observe the available restriction enzymes in the different input variations (Fig. 6).

#### Retrieval of variation flanking sequences

To improve the efficiency in searching for recognition sequences in different alleles, REHUNT implements a flanking sequence retrieval function, which is centered by a variation. It is proposed to be able to compress the sequence length. Shorter sequences can capture restriction enzymes rapidly, and longer sequence may take much longer to align recognition sequences to obtain the restriction enzymes.

#### Commercial and non-commercial enzymes

More and more restriction enzymes are being discovered in biological and medical research. However, not all restriction enzymes are available for experiments. Commercial enzymes are easily obtained. Many companies provide commercial restriction enzymes, including Takara, Boehringer Mannheim, and New England Biolabs. REHUNT is able to distinguish the source of the restriction enzymes to help researchers obtain them for their experiments.

#### Composite IUPAC and general enzymes

Composite IUPAC enzymes usually provide a combination of recognition sequences, allowing them to recognize other positions, while general restriction enzymes only recognize one recognition sequence. REHUNT provides a classification of composite IUPAC and general enzymes for researchers.

## Results and discussion

REHUNT is proposed to solve restriction enzymes-based manipulations for all kinds of biological applications. The following shows the environment, characteristics, materials and methods, examples for REHUNT, and availability and update frequency.

### Characteristics

REHUNT accepts inputs, including regular DNA sequence, variation-in-sequence indicated by IUPAC code, as well as variation-in-sequence indicated by dNTPs format. Unlike other restriction enzymes search tools, REHUNT not only can identify di-allelic variation, i.e. [dNTP1/dNTP2], but also tri-, tetra-allelic and indel-allelic variations, i.e. [dNTP1/dNTP2/dNTP3], [dNTP1/dNTP2/dNTP3/dNTP4], and [−/dNTPn]. REHUNT is capable of providing the available restriction enzymes to distinguish variant alleles for RFLP analysis in a sequence on both the sense and anti-sense strands. Variant-alleles with restriction enzymes-available or -unavailable, i.e. RFLP enzyme availability, can be judged using the class of “JudgeRFLP”. The length of the restriction recognition sequence, as well as the restriction recognition sequences with composite IUPAC or general sequence format, can be assigned for the output. All hunted restriction enzymes are provided in four categories relying on the class of “EnzymeClassification”, including composite IUPAC, general sequence (‘A’, ‘T’, ‘G’ and ‘C’ nucleotide only), commercial and non-commercial types. Information for every searched enzyme name can be obtained from their REBASE entry using the class of “REBASE”. Furthermore, REHUNT is capable of performing high throughput RFLP analysis using the JAVA thread function in the class of “JudgeRFLPBatchThread”. Examples of all kinds of manipulations in REHUNT are available.

### Materials and methods

**Fig. 7:**
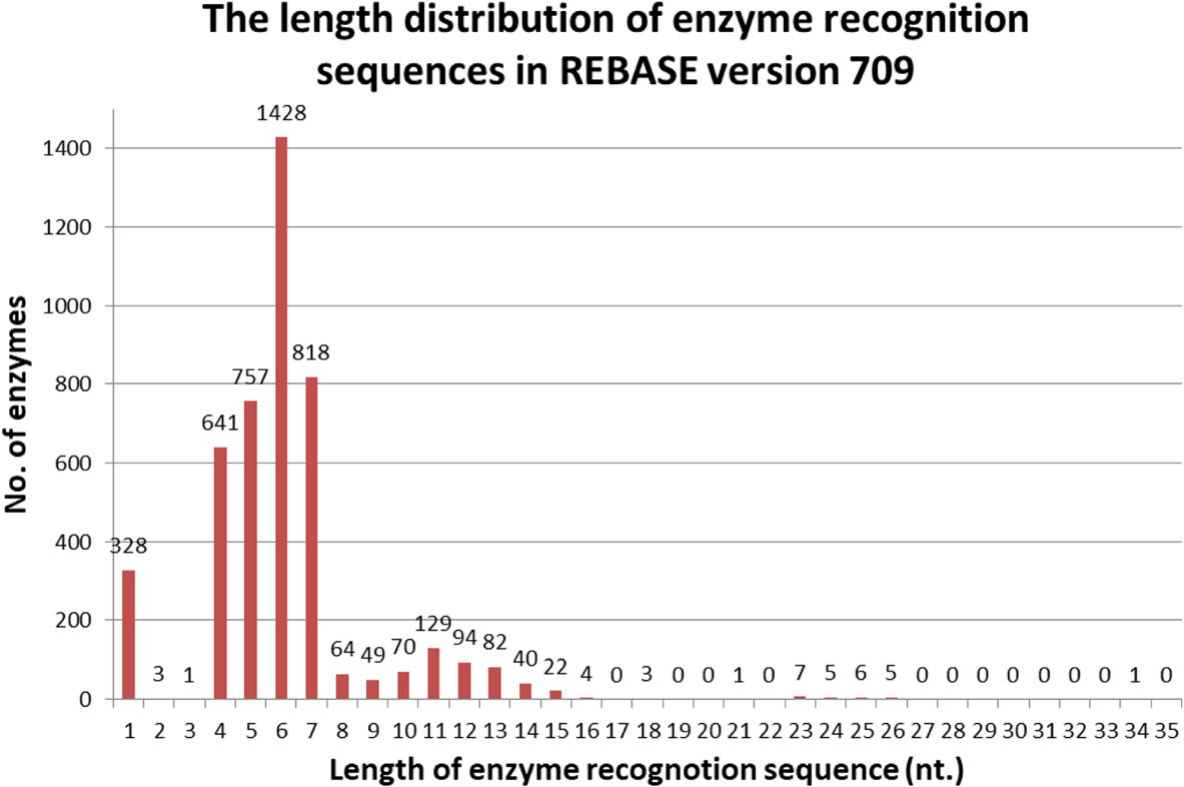
The length distribution of enzyme recognition sequences in REBASE version 709

**Fig. 8:**
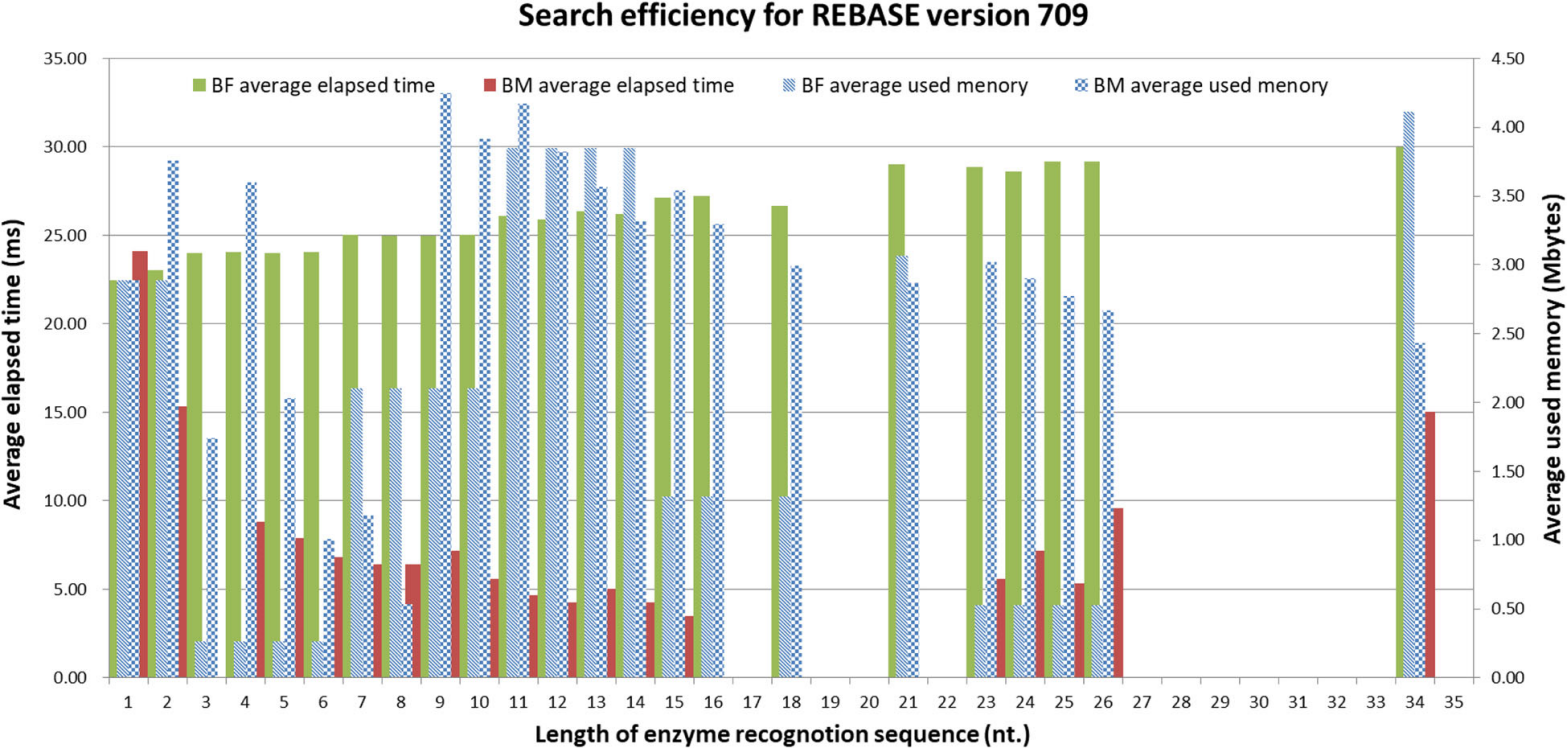
Search efficiency for REBASE version 709

REHUNT has been developed over many years. All the functions have been tested and verified, and bugs have been corrected. It is stable and reliable. The latest test of REHUNT comprised a search for restriction enzymes based on a high throughput analysis of 381 SNPs. These SNPs are found in the gene SLC6A4, and were filtered without merged SNPs and are represented in alleles type with 500 bps of SNP flanking sequence is retrieved by SNP-Flankplus [21]. The dataset (SLC6A4_Alleles_381SNPs.txt) is provided at https://sites.google.com/site/yhcheng1981/rehunt. Furthermore, the search efficiency, including elapsed time and used memory, were also tested and compared with traditional search methods, i.e. Brute-Force based on REBASE version 709. A total of 4558 enzyme recognition sequences with different lengths from REBASE have been tested on an Intel(R) Core(TM) i7-4700HQ CPU @ 2.40 GHz × 2, and 8GB of RAM under Windows 10 64 bits. The length distribution of the enzyme recognition sequences for REBASE version 709 is shown in Fig. 7. The search result for average elapsed time (ms) and average used memory (Mbytes) are shown in Fig. 8. Table 2 shows the values for the average elapsed time and average memory used. Furthermore, the ratios between Brute-Force and Boyer-Moore is also shown. Table 2 shows that the search method in REHUNT is very efficient, both in terms of on time and memory compared with the traditional Brute-Force method. The test program (TestEnzymeSearch.jar) and its user manual can be obtained at https://sites.google.com/site/yhcheng1981/rehunt. It is worth mentioning that REHUNT has been integrated into a practical method for natural PCR-RFLP primer design for SNP genotyping [12].

**Table 2:** The comparison of the search efficiency of the Brute Force and Boyer-Moore methods for different lengths of enzyme recognition sequences based on REBASE version 709. Brute Force is presented as “BF”; Boyer-Moore is presented as “BM”

Enzyme recognition sequence length	Enzyme recognition sequence number	Average elapsed time (ms)		The elapsed time ratio in BF/BM	Average used memory (Mbytes)		The used memory ratio in BF/BM
		BF	BM		BF	BM	
1	328	22.45	24.09	0.93	2.89	2.89	1.00
2	3	23.00	15.33	1.50	2.89	3.75	0.77
3	1	24.00	0.00	–	0.26	1.74	0.15
4	641	24.03	8.80	2.73	0.26	3.59	0.07
5	757	24.00	7.91	3.04	0.26	2.03	0.13
6	1428	24.06	6.80	3.54	0.26	1.00	0.26
7	818	25.01	6.41	3.90	2.10	1.18	1.79
8	64	24.95	6.39	3.90	2.10	0.53	3.95
9	49	24.96	7.16	3.48	2.10	4.24	0.50
10	70	25.03	5.60	4.47	2.10	3.91	0.54
11	129	26.08	4.66	5.60	3.85	4.17	0.92
12	94	25.87	4.24	6.10	3.85	3.82	1.01
13	82	26.37	5.04	5.23	3.85	3.56	1.08
14	40	26.20	4.28	6.13	3.85	3.31	1.16
15	22	27.14	3.50	7.75	1.31	3.54	0.37
16	4	27.25	0.00	–	1.31	3.29	0.40
17	0	0.00	0.00	–	0.00	0.00	–
18	3	26.67	0.00	–	1.31	2.99	0.44
19	0	0.00	0.00	–	0.00	0.00	–
20	0	0.00	0.00	–	0.00	0.00	–
21	1	29.00	0.00	–	3.06	2.86	1.07
22	0	0.00	0.00	–	0.00	0.00	–
23	7	28.86	5.57	5.18	0.53	3.02	0.17
24	5	28.60	7.20	3.97	0.53	2.90	0.18
25	6	29.17	5.33	5.47	0.53	2.77	0.19
26	5	29.20	9.60	3.04	0.53	2.67	0.20
27	0	0.00	0.00	–	0.00	0.00	–
28	0	0.00	0.00	–	0.00	0.00	–
29	0	0.00	0.00	–	0.00	0.00	–
30	0	0.00	0.00	–	0.00	0.00	–
31	0	0.00	0.00	–	0.00	0.00	–
32	0	0.00	0.00	–	0.00	0.00	–
33	0	0.00	0.00	–	0.00	0.00	–
34	1	30.00	15.00	2.00	4.11	2.43	1.69
35	0	0.00	0.00	–	0.00	0.00	–

### Examples for REHUNT

REHUNT provides six examples for user operations. Users can refer to and modify the examples for their preferred applications. The examples are shown in Table 3. Furthermore, in order to help users to actually try it, one of intuitive ways running in windows 64-bit operating system is provided to guide users to perform the examples. The guideline can be downloaded from https://sites.google.com/site/yhcheng1981/rehunt.

**Table 3:** Six examples of REHUNT provided for user operations

Example ID	Function	Description
1	Universal judgement of whether a variation of a sequence can be recognized by restriction enzymes	The input variations may include two variations, three variations, or four variations.
2	Search for specific restriction enzymes	For example, the sequence “ACGG[A/C]TTTTTT” can be recognized by restriction enzyme TspGWI (ACGGA) for variation A. The sequence “ACGG[A/C]TTTTTTACGGATTT” can be recognized by restriction enzyme TspGWI (ACGGA) for variation A, but it will be excluded because of the repeat of the sequence “ACGGA”. REHUNT identifies specific and available restriction enzymes, thus, the reappearing restriction enzyme TspGWI (ACGGA) will be excluded.
3	Identify restriction enzymes for a sequence and complementary sequence with multi-variation (Please use Example 3_1, Example 3_2, and Example 3_3)	The sequence “TTAGCATCAGCATTTGCTGC[multi-variation] ATCGCTAACGGTGGATCTAC” with multi-variation that can be recognized by restriction enzymes. Its complementary sequence can also be recognized by restriction enzymes. These restriction enzymes are easy identified by REHUNT. Example 3_1, Example 3_2, and Example 3_3 are provided for two variations, three variations, and four variations, respectively.
4	Only restriction enzymes with eliminated IUPAC format are evaluated	The sequence “AATTTCTGG[A/G]CCCTAACGGT” can only be recognized by restriction enzyme BspGI (CTGGAC) with eliminated IUPAC format for variation A. The function setIUPACenzyme(false) in “JudgeRFLP” class is used.
5	All restriction enzymes including IUPAC format are evaluated	The sequence “AATTTCTGG[A/G]CCCTAACGGT” can be recognized by many restriction enzymes including IUPAC format for variation A. The function setIUPACenzyme(true) in “JudgeRFLP” class is used.
6	High throughput analysis	The multiple sequences can be analyzed by “JudgeRFLPBatchThread” class. The function is useful for high throughput analysis.

### Availability and update frequency

REHUNT has been under development since 2007. It has been continually verified and tested for stability, availability, and flexibility. It is now ready for release to provide the better biological applications. The REHUNT source code and its API documents are accessible at SourceForge: https://sourceforge.net/projects/rehunt/, GitHub: https://github.com/yuhuei/rehunt, and at: https://sites.google.com/site/yhcheng1981/rehunt. The package is implemented in JAVA and it can be robustly integrated into other software and methods. The REBASE version [8] is updated annually and built into a local database. We suggest that considering the rapid update frequency of REBASE version, users should update their version manually.

All academic researchers are encouraged to use REHUNT in their studies or to integrate it into their systems and applications. Non-academic users or commercial needs are also welcome to use it. For further information or additional applications, please contact the author Yu-Huei Cheng <yuhuei.cheng@gmail.com>.

REHUNT implement various methods including sequence processing, filtering, contents comparison, restriction enzyme information retrieval, and RFLP analysis; therefore, to facilitate researchers to integrate their applications with REHUNT, helpful API documents (REHUNT_v1.2_doc.zip) for all the methods used in REHUNT are provided for the users and developers.

## Conclusions

REHUNT is freely available and is based on a GPL v3 license. It has been developed as a convenient, reliable, efficient, and flexible package. It provides a powerful enzyme hunting tool for RFLP genotyping and simultaneously supports high throughput analysis for batch sequences. The highlighted features include: 1) Quick search for restriction enzymes throughout a sequence based on the Boyer-Moore algorithm; 2) all available restriction enzymes provided and regularly updated from REBASE; 3) an open source API is available to integrate with all kinds of bioinformatics systems and applications; 4) SNP genotyping is available for plant and animal marker-assisted breeding, as well as for human genetics; and 5) high throughput analysis is available for Next Generation Sequencing (NGS). REHUNT is not only effective to identify for restriction enzymes sites in a sequence but also is available for SNP genotyping. SNPs are associated with important agricultural and medical phenotypes, including sensitivity to disease or environmental stress, and response to drugs or treatment regimens. Therefore, SNP genotyping can be used for plant and animal marker-assisted breeding programs to accelerate selective breeding, which saves time and money. In addition, SNP genotyping can be applied to large-scale epidemiological studies to identify specific variations that affect susceptible to disease and to drug or therapeutic responses. Other applications such as genealogy studies, precision medicine, personalized medical care, and genetic fingerprint can be analyzed by REHUNT. REHUNT offers useful RFLP information for cost-effective association studies. Advanced users and developers can use, integrate, and modify the code for their research and applications.

## Abbreviations

API: Application programming interface
BNDM: Backward non-deterministic Dawg matching
BOM: Backward oracle matching
IUPAC: International union of pure and applied chemistry chemical nomenclature
KMP: Knuth-Morris-Pratt
NGS: Next generation sequencing
OOP: Object-oriented programming
REBASE: Restriction enzyme database
REHUNT: Restriction enzymes HUNTing
RFLP: Restriction fragment length polymorphism
SNP: Single Nucleotide polymorphism
